# Authors’ Response to Peer Reviews of “Challenges in Implementing a Mobile AI Chatbot Intervention for Depression Among Youth on Psychiatric Waiting Lists: Randomized Controlled Study Termination Report”

**DOI:** 10.2196/82083

**Published:** 2025-09-05

**Authors:** Junichi Fujita, Yuichiro Yano, Satoru Shinoda, Noriko Sho, Masaki Otsuki, Akira Suda, Mizuho Takayama, Tomoko Moroga, Hiroyuki Yamaguchi, Mio Ishii, Tomoyuki Miyazaki

**Affiliations:** 1 Department of Child Psychiatry, Yokohama City University Hospital, 3-9, Fukuura, Kanazawa-ku, Yokohama, 236-0004, Japan, 81 0457872800; 2 Psychiatric Center, Yokohama City University Medical Center, Yokohama, Japan; 3 Department of Biostatistics, Yokohama City University School of Medicine, Yokohama, Japan; 4 Kanagawa Children's Medical Center, Yokohama, Japan; 5 Fujisawa City Hospital, Fujisawa, Japan; 6 Department of Psychiatry, Yokohama City University School of Medicine, Yokohama, Japan; 7 Center for Promotion of Research and Industry-Academic Collaboration, Yokohama City University, Yokohama, Japan

**Keywords:** randomized controlled trial, AI chatbot, acceptance and commitment therapy, mental health, psychiatry, children, adolescents, Japan

*This is the authors’ response to peer-review reports for “Challenges in Implementing a Mobile AI Chatbot Intervention for Depression Among Youth on Psychiatric Waiting Lists: Randomized Controlled Study Termination Report.”*

## Round 1 Review

### Reviewer E [1]

*Thank you for inviting me to review this paper [2]. However, my suggestion would be that this paper should be rejected. I am very cognizant of publication biases, and I am a firm believer that the publication of negative results is very important. I therefore have no problem with the fact that the sample decreased to zero. However, I do believe that more detail is needed in terms of why people disengaged. The rationale for the paper is set up as efficacy of the intervention, but the main message of the paper is that the sample declined. I would therefore like more emphasis on qualitative interviews that examined why people disengaged. Follow-up work such as this would make a very interesting paper.*

**Response:** We appreciate this insightful comment. While our study did not initially include structured qualitative interviews, we recognize that a more systematic approach to understanding disengagement barriers would provide deeper insights. In response, we have revised the Strengths and Limitations section in the Discussion to explicitly acknowledge this limitation and propose the incorporation of structured exit interviews or surveys in future studies. Additionally, we have expanded the Results section to include details of participant withdrawal, particularly emphasizing parental reports of the adolescent’s distress regarding online participation.

### Reviewer I [3]

#### General Comments

*This paper describes the results of a parallel group randomized controlled trial that examined the feasibility of an artificial intelligence (AI) chatbot-led mental health intervention to support pediatric patients on the psychiatry waitlists in Japan. The article is well-written and organized, and the objectives of the study are clearly stated. Methodology elements such as eligibility criteria, information sources, and data collection process are clear. A clear list of outcomes and variables for which data were researched is presented. The authors provide an important contribution to the field by reporting on factors that challenge adolescents’ engagement in digital mental health interventions and providing meaningful recommendations for future research.*

#### Specific Comments

##### Major Comments

*1. How many chatbots were shortlisted, and why was emol favored over the others, given the selection criteria? (Under AI Chatbot Selection Process.)*

**Response:** We have clarified the AI Chatbot Selection Process section, detailing that multiple AI chatbots were reviewed based on predefined selection criteria. AI chatbot emol was chosen due to its integration of acceptance and commitment therapy (ACT) principles, user engagement features, and prior application in mental health settings.

*2. How are the six core processes of ACT delivered in the AI chatbot (under Intervention Group)? Expand more on each section. How does the session meet the core processes of ACT—acceptance, cognitive defusion, being present, self as context, values, and committed action?*

**Response:** We have expanded the Intervention Group section to describe how each ACT process—acceptance, cognitive defusion, being present, self-as-context, values, and committed action—is incorporated into specific chatbot sessions. Additionally, we have created a supplementary table that provides a comprehensive overview of the session structure, showing how each session aligns with specific ACT processes. The table details the content types (videos, comics, written practices) used to deliver these therapeutic concepts in an engaging and accessible format for adolescents.

*3. How was the section structured? Did adolescents go through modules? Could they write anything to the chatbot, or was the content predefined? Were the sessions sequentially delivered or not? Could they access previously completed modules or track their progress?*

**Response:** The revised Intervention Group section now clarifies session progression, user interaction (predefined vs open-ended responses), and module accessibility. We have also created a supplementary figure illustrating the actual interface of the emol application, including screenshots of conversations between the AI character Roku and users. This figure demonstrates how therapeutic concepts are introduced in a conversational age-appropriate manner, and how users engage with the app through both structured exercises and dialogues.

*4. Were there any safeguarding links and referral contacts built into the chatbot in case participants needed additional support beyond those offered by the chatbot? If yes, I recommend including it under the ethics paragraph.*

**Response:** We have added details in the Ethical Considerations section, confirming that emergency support information was provided to all participants and that the research team had a protocol for directing participants to appropriate psychiatric services if necessary.

*5. How were you planning to investigate engagement? Would you report on the frequency of use, number of interactions with the chatbot, or amount of content visualized by participants? Even though the study’s main questions are not focused on engagement, I suggest that the authors consider including an engagement outcome paragraph right after the secondary outcomes.*

**Response:** The Data Management section now specifies that engagement was tracked through AI chatbot emol’s data logs, recording total usage time, average daily usage, session progression, and last completed session. These engagement metrics were intended to assess both usage patterns and their relationship with depressive symptom changes.

##### Minor Comments

*6. I recommend moving all hyperlinks to the appendix and including an image of the chatbot. I also recommend that authors include an image of the intervention delivered through the hospital website.*

**Response:** We have relocated hyperlinks to the appendix and created a supplementary figure showing the chatbot interface and interaction examples. The figure includes screenshots of the AI character Roku and demonstrates key features of the app, including how it introduces ACT concepts, guides users through exercises, and provides supportive feedback. This visual representation helps clarify the user experience and the app’s design elements specifically tailored for adolescents.

*7. Please state the statistical methods used to deal with missing data.*

**Response:** The Statistical Analysis section now explicitly states the methods used to handle missing data. Missing data were analyzed as observed without imputation, with the primary analysis performed on the full analysis set. Given the exploratory nature of this study and the lack of prior research in this specific population, no imputation was conducted, ensuring that the results accurately reflect the available data without introducing assumptions through imputation methods.

*8. In the Discussion, you argue that young people prefer online mental health support over in-person support [4]. I believe you could discuss this a bit more in your Introduction paragraph to strengthen your discussion regarding the potential gap online services could fill.*

**Response:** The Introduction section has been revised, particularly in the first paragraph, to incorporate a discussion on the preference for online mental health support among youth. This revision, supported by existing literature, emphasizes the increasing demand for accessible and scalable digital mental health interventions.

*9. I recommend including a paragraph under the Introduction on previous Japanese studies focusing on chatbot-led or digital mental/public health interventions to provide an overview of the current population uptake of digital health interventions.*

**Response:** A paragraph summarizing prior chatbot-led interventions in Japan has been added to the Introduction section, specifically in the fifth paragraph. This revision also incorporates cultural factors unique to Japan, providing a more comprehensive understanding of how chatbot interventions are perceived and utilized in this context.

### Reviewer M [5]

#### General Comments

*The topic and objectives of the study are certainly interesting, as depression among young individuals is an increasingly pervasive and growing problem globally, exacerbated by the COVID-19 pandemic, as the authors themselves point out. Furthermore, the use of AI to support traditional methods of treating this condition makes the study topical. The paper is well-written and comprehensible throughout; the supporting bibliography is adequate; it has a good methodological approach, with clear and well-defined objectives, and an accurate description of the inclusion and exclusion criteria for participants. Although the statistical analyses planned by the authors are consistent with the objectives they have defined, the lack of availability of data on which to carry out these analyses and, therefore, the absence of results does not allow an evaluation of this specific aspect. However, the authors have posited potential explanations for instances of nonadherence to the intervention protocol, which are substantiated by extant literature on the subject, therefore apprising the reader of the possible limitations of this type of intervention in this specific population that fulfills certain inclusion criteria. The paper thus provides a cue and guidance for future studies in this field. Lastly, as stated in the major comments below, the major shortcoming of this study is the lack of clarity as to whether the authors used an active or nonactive control group.*

#### Specific Comments

##### Major Comments

*1. In the Study Design paragraph, the authors stated that the control group would receive standard care (making it an active control group), while in the Control Group paragraph, they stated that they would receive general mental health information and would undergo online evaluations and diary recordings (making it a nonactive control group). It is not clear if the authors deem these two procedures similar. In the event that they do not regard them as analogous, it would be beneficial to ascertain which of the two would have been delivered to the control group. Furthermore, it would be appreciated if the authors could provide an explanation and make the appropriate adjustments in the manuscript about (1) what standard care would have comprised and (2) what is the nature of the short video programs that participants received as general mental health information, in order to enable the reader to ascertain whether they are informational videos, mental health support videos, etc.*

**Response:** We have revised the Study Design and Control Group sections to clarify that the control group received general mental health information via a publicly available website, not standard psychiatric care. The educational materials include child-friendly videos featuring a psychiatrist explaining mental health topics using animated characters.

Additionally, the Introduction (fifth paragraph) now provides a more detailed discussion of prior chatbot-led interventions in Japan. The Methods section has been expanded to specify the content of the videos and the online evaluations used in the control condition, including voice analysis and writing pressure measurements, ensuring transparency in the study’s evaluation process.

## Round 2 Review

### Reviewer M

#### General Comments

*I would like to express my gratitude to the authors for implementing the requested revisions, which have served to enhance the clarity and thoroughness of the manuscript. Still, there are some elements that, in my view, would benefit from modification.*

#### Specific Comments

##### Major Comments

*1. Supplementary Table 1 and the supplementary figure are missing.*

**Response:** We apologize for the oversight. We have now uploaded Supplementary Table 1 and the supplementary figure as separate multimedia appendix files and referenced them in the manuscript as “Multimedia Appendix 1” and “Multimedia Appendix 2,” respectively.

*2. The sentence “AI chatbot emol features a friendly character name ‘Roku’” is redundant, as the same concept is repeated in the preceding sentence (in the AI Chatbot Selection Process paragraph).*

**Response:** Thank you for pointing this out. We have removed the redundant sentence “AI chatbot emol features a friendly character named ‘Roku,’ who guides users through ACT-based conversations in a relatable manner.” to improve conciseness.

Before: “AI chatbot emol’s design prioritizes accessibility and engagement, particularly for young users, by featuring a friendly AI character named ‘Roku.’ AI chatbot emol features a friendly character named ‘Roku,’ who guides users through ACT-based conversations in a relatable manner.”

After: “AI chatbot emol’s design prioritizes accessibility and engagement, particularly for young users, by featuring a friendly AI character named ‘Roku.’”

*3. The following sentence is repeated twice: “Weekly online assessments were conducted at Week 0, during the intervention period, and at Week 9” (in the Intervention Group paragraph).*

**Response:** Thank you for noting the repetition. We have removed the duplicated sentence to streamline the narrative.

Before: “Weekly online assessments were conducted at Week 0, during the intervention period, and at Week 9. Weekly online assessments were conducted at Week 0, during the intervention period, and at Week 9.”

After: “Weekly online assessments were conducted at Week 0, during the intervention period, and at Week 9.”

*4. The sentence “Non physician research assistants encouraged participants to use the pen consistently for their diary entries and performed minimal mental status checks during these assessments” is redundant, as the same concept is repeated afterward in the same paragraph (Intervention Group section). Therefore, it should be deleted to streamline the text.*

**Response:** We agree with the reviewer and have removed the redundant sentence about nonphysician research assistants encouraging diary use and conducting minimal mental status checks.

Before: “Weekly online assessments were conducted at Week 0, during the intervention period, and at Week 9. Non physician research assistants encouraged participants to use the pen consistently for their diary entries and performed minimal mental status checks during these assessments.”

After: “Weekly online assessments were conducted at Week 0, during the intervention period, and at Week 9.”

*5. In what manner was the viewing of the videos organized for the control group? Was a schedule in place, or were the participants free to watch the videos at their own discretion? Furthermore, how was the actual viewing of the videos ascertained?*

**Response:** Thank you for this important question. We have revised the Methods section to clarify that while there was no formal schedule imposed for video viewing, research assistants did confirm and record whether participants had viewed the assigned video content during each weekly assessment. The sentence “Participants were free to view the videos at their own discretion, without a predefined schedule. However, research assistants confirmed and recorded whether participants had viewed the assigned video content during each assessment session.” was added in the Control Group section.

Before: “The control group received general mental health information via the Yokohama City University child psychiatry department’s website, ‘Oyako-no Kokoro-no Tomarigi’ (Appendix). This website provides educational resources about common mental health conditions in children and adolescents through easy-to-understand videos and text explanations specifically designed for young people. The video content features conversations between teddy bear and rabbit avatars discussing common mental health symptoms and concerns in children and adolescents, followed by child-friendly explanations from a child psychiatrist. Topics covered in these educational videos include: suicidal thoughts, lack of energy/motivation, anxiety, isolation and loneliness, obsessive worrying, attention difficulties, self-harm behaviors, sleep problems, and auditory hallucinations. The child psychiatrist appearing in these videos is one of the authors of this study (JF). The website also contains separate sections with mental health resources for children and families, including multiple Q&A entries about children’s mental health issues. These materials are purely informational and educational in nature, rather than providing interactive or personalized therapeutic interventions. Participants were free to view the videos at their own discretion, without a predefined schedule. However, research assistants confirmed and recorded whether participants had viewed the assigned video content during each assessment session.”

After: “The control group received general mental health information via the Yokohama City University child psychiatry department’s website, ‘Oyako-no Kokoro-no Tomarigi’ (Appendix). This website provides educational resources about common mental health conditions in children and adolescents through easy-to-understand videos and text explanations specifically designed for young people. The video content features conversations between teddy bear and rabbit avatars discussing common mental health symptoms and concerns in children and adolescents, followed by child-friendly explanations from a child psychiatrist. Topics covered in these educational videos include: suicidal thoughts, lack of energy/motivation, anxiety, isolation and loneliness, obsessive worrying, attention difficulties, self-harm behaviors, sleep problems, and auditory hallucinations. The child psychiatrist appearing in these videos is one of the authors of this study (JF). The website also contains separate sections with mental health resources for children and families, including multiple Q&A entries about children’s mental health issues. These materials are purely informational and educational in nature, rather than providing interactive or personalized therapeutic interventions. Participants were free to view the videos at their own discretion, without a predefined schedule. However, research assistants confirmed and recorded whether participants had viewed the assigned video content during each assessment session.”

*6. In my personal view, the use of an active control group would have been a valuable approach, for instance, by comparing two distinct chatbots providing different types of therapy, the evaluation of which would have determined which one would prove to be more efficacious in terms of symptoms improvement. This approach would have ensured that both groups received a therapeutic intervention and could have provided additional information in terms of engagement and usability. The authors stated that the design they chose “reflects the real-world experience of many psychiatric waiting list patients in Japan,” but as they also declared, “the lack of timely intervention can exacerbate symptoms and increase the risk of severe outcomes.” Therefore, given such a risk, my question is: what is the rationale behind the authors’ decision to employ a passive control group?*

**Response:** Thank you for this thoughtful suggestion. We agree that an active control group, such as a comparison between two therapeutic chatbots, could offer richer insights regarding engagement and efficacy.

However, our extensive prestudy evaluation revealed that emol was the only app meeting all essential criteria: (1) evidence-based therapeutic framework (ACT), (2) age-appropriate design for adolescents, (3) availability for clinical research, and (4) cost-effective access for research purposes. We conducted systematic reviews of Japanese mental health apps and interviewed multiple developers, but no comparable alternative was identified that met these combined requirements. No other chatbot meeting these criteria was identified during our review. Therefore, we adopted a passive control condition to reflect the current standard experience for patients on psychiatric waiting lists in Japan, where no structured digital intervention is provided. We have added a clarification: “While an active control group could have offered more rigorous comparison, we selected a passive control condition due to practical constraints. At the time of study planning, emol was the only adolescent-appropriate AI chatbot in Japan that integrated evidence-based psychological content (ACT), had a suitable user interface, and was available for research use. No other comparable tool was identified. Thus, we chose a passive control to reflect the real-world conditions in Japan, where patients on psychiatric waiting lists typically receive only basic informational support.” in the Discussion section.

Before: “This study may have unintentionally targeted a population less receptive to alternative digital interventions. Families who had already secured an upcoming psychiatric appointment may have seen little value in participating in a study involving digital interventions, preferring instead to wait for their scheduled in-person consultation. For these families, traditional in-person care may have appeared more reassuring, especially given the severity of the patient’s symptoms. Previous research on social influences in mental health service-seeking behavior among young people suggests that family is often the primary influence in choosing in-person services, whereas young people themselves tend to make decisions regarding online services [4]. Another study has also found that parents often seek informal support for their children’s mental health concerns initially, only turning to professional services as issues become more severe [6]. Additionally, patients with severe symptoms or their families often prefer in-person consultations over digital interventions, perceiving in-person care as more reliable and suitable for managing serious symptoms [7]. Therefore, patients and families may value the familiarity and perceived efficacy of traditional, in-person care as a more reliable or reassuring option compared to digital alternatives. This preference likely contributed to the reluctance toward digital solutions observed in this study. Engaging patients and families earlier in the mental health care process—before they have secured traditional clinical appointments—might improve receptiveness to digital options. While an active control group could have offered more rigorous comparison, we selected a passive control condition due to practical constraints. At the time of study planning, emol was the only adolescent-appropriate AI chatbot in Japan that integrated evidence-based psychological content (ACT), had a suitable user interface, and was available for research use. No other comparable tool was identified. Thus, we chose a passive control to reflect the real-world conditions in Japan, where patients on psychiatric waiting lists typically receive only basic informational support.”

After: “This study may have unintentionally targeted a population less receptive to alternative digital interventions. Families who had already secured an upcoming psychiatric appointment may have seen little value in participating in a study involving digital interventions, preferring instead to wait for their scheduled in-person consultation. For these families, traditional in-person care may have appeared more reassuring, especially given the severity of the patient’s symptoms. Previous research on social influences in mental health service-seeking behavior among young people suggests that family is often the primary influence in choosing in-person services, whereas young people themselves tend to make decisions regarding online services [4]. Another study has also found that parents often seek informal support for their children’s mental health concerns initially, only turning to professional services as issues become more severe [6]. Additionally, patients with severe symptoms or their families often prefer in-person consultations over digital interventions, perceiving in-person care as more reliable and suitable for managing serious symptoms [7]. Therefore, patients and families may value the familiarity and perceived efficacy of traditional, in-person care as a more reliable or reassuring option compared to digital alternatives. This preference likely contributed to the reluctance toward digital solutions observed in this study. Engaging patients and families earlier in the mental health care process—before they have secured traditional clinical appointments—might improve receptiveness to digital options. While an active control group could have offered more rigorous comparison, we selected a passive control condition due to practical constraints. At the time of study planning, emol was the only adolescent-appropriate AI chatbot in Japan that integrated evidence-based psychological content (ACT), had a suitable user interface, and was available for research use. No other comparable tool was identified. Thus, we chose a passive control to reflect the real-world conditions in Japan, where patients on psychiatric waiting lists typically receive only basic informational support.”

*7. The concept expressed in the sentence “Another patient refused participation due to concerns about the diary entry, and the third patient was excluded after starting therapy at another facility” is also conveyed in the preceding sentence (in the Results paragraph). It is recommended that one of the two sentences be deleted.*

**Response:** We thank the reviewer for identifying the redundancy in our description of patient enrollment. We agree that the two sentences contained overlapping information about the same two patients. The sentence, “Another patient declined participation due to concerns about diary recording, and the third patient was excluded after beginning medication at another facility.” was removed.

Before: “Among the three patients who completed the informed consent process, one participant (a female adolescent) provided consent but subsequently withdrew from the study. The participant’s family initially contacted the research team on the scheduled day of the first online session, stating: ‘This morning, she became panic-stricken and is now unable to participate. Although it is the day of the appointment, would it be possible to cancel? I sincerely apologize for the inconvenience caused after all your preparations.’ In a follow-up message, the family elaborated: ‘She expressed anxiety about the online interview, making it impossible to proceed. We had hoped that engaging in this activity might help her develop a more positive outlook, but perhaps it was still too challenging for her.’ The other two patients who completed the informed consent process either declined participation due to concerns about diary recording requirements or were excluded after beginning medication at another facility. Another patient declined participation due to concerns about diary recording, and the third patient was excluded after beginning medication at another facility. Consequently, no evaluable data were obtained in this study.”

After: “Among the three patients who completed the informed consent process, one participant (a female adolescent) provided consent but subsequently withdrew from the study. The participant’s family initially contacted the research team on the scheduled day of the first online session, stating: ‘This morning, she became panic-stricken and is now unable to participate. Although it is the day of the appointment, would it be possible to cancel? I sincerely apologize for the inconvenience caused after all your preparations.’ In a follow-up message, the family elaborated: ‘She expressed anxiety about the online interview, making it impossible to proceed. We had hoped that engaging in this activity might help her develop a more positive outlook, but perhaps it was still too challenging for her.’ The other two patients who completed the informed consent process either declined participation due to concerns about diary recording requirements or were excluded after beginning medication at another facility. Consequently, no evaluable data were obtained in this study.”

## Abbreviations

ACT: acceptance and commitment therapy
AI: artificial intelligence
